# Productivity Loss Related to Neglected Tropical Diseases Eligible for Preventive Chemotherapy: A Systematic Literature Review

**DOI:** 10.1371/journal.pntd.0004397

**Published:** 2016-02-18

**Authors:** Edeltraud J. Lenk, William K. Redekop, Marianne Luyendijk, Adriana J. Rijnsburger, Johan L. Severens

**Affiliations:** 1 Institute of Health Policy and Management, Erasmus University Rotterdam, Rotterdam, The Netherlands; 2 Medical Delta, Delft, The Netherlands; Australian National University, AUSTRALIA

## Abstract

**Background:**

Neglected Tropical Diseases (NTDs) not only cause health and life expectancy loss, but can also lead to economic consequences including reduced ability to work. This article describes a systematic literature review of the effect on the economic productivity of individuals affected by one of the five worldwide most prevalent NTDs: lymphatic filariasis, onchocerciasis, schistosomiasis, soil-transmitted helminths (ascariasis, trichuriasis, and hookworm infection) and trachoma. These diseases are eligible to preventive chemotherapy (PCT).

**Methodology/Principal Findings:**

Eleven bibliographic databases were searched using different names of all NTDs and various keywords relating to productivity. Additional references were identified through reference lists from relevant papers. Of the 5316 unique publications found in the database searches, thirteen papers were identified for lymphatic filariasis, ten for onchocerciasis, eleven for schistosomiasis, six for soil-transmitted helminths and three for trachoma. Besides the scarcity in publications reporting the degree of productivity loss, this review revealed large variation in the estimated productivity loss related to these NTDs.

**Conclusions:**

It is clear that productivity is affected by NTDs, although the actual impact depends on the type and severity of the NTD as well as on the context where the disease occurs. The largest impact on productivity loss of individuals affected by one of these diseases seems to be due to blindness from onchocerciasis and severe schistosomiasis manifestations; productivity loss due to trachoma-related blindness has never been studied directly. However, productivity loss at an individual level might differ from productivity loss at a population level because of differences in the prevalence of NTDs. Variation in estimated productivity loss between and within diseases is caused by differences in research methods and setting. Publications should provide enough information to enable readers to assess the quality and relevance of the study for their purposes.

## Author Summary

Neglected Tropical Diseases (NTDs) not only have impact on health and life expectancy of mostly disadvantaged populations, but can also lead to economic consequences, including reduced ability to work. Investments in health improvement of the populations affected by NTDs would also help to increase economic growth of the affected regions, since healthier populations are more economically productive. We performed a systematic literature review to better understand how much NTDs affect people’s economic welfare. Here we present the results for the NTDs that are controlled with preventive chemotherapy (PCT): lymphatic filariasis, onchocerciasis, schistosomiasis, soil-transmitted helminths (ascariasis, trichuriasis, and hookworm infection) and trachoma. Our findings show that PCT NTDs clearly affect productivity, although the actual impact depends on the type and severity of the NTD as well as on the context where the disease occurs. Variation in estimated productivity loss is also caused by differences in research methods. Publications should provide enough information to enable readers to assess the quality and relevance of the study for their purposes.

## Introduction

Most of the people affected by Neglected Tropical Diseases (NTDs) are impoverished and marginalized populations, with low visibility and little political voice. They are not considered a priority market for pharmaceutical manufacturers or a health risk for the wealthier parts of the world. [1–3] Nevertheless, NTDs have an important impact on child development, school attendance, learning, nutritional status, pregnancy outcomes, and worker productivity, especially in poor rural settings, where physical labor is the major subsistence mode. As any other disease, they can lead to productivity loss in many ways, including reduced productivity at work (presenteeism), absence from work (absenteeism) or even job loss, depending on the type, severity and duration of the disease. [2–12]

Many publications in the literature describe the epidemiological and physical aspects of NTDs. In contrast, the impact of NTDs on paid and unpaid work and the productivity of individual men and women has been less frequently studied. Most of the data about the economic burden of NTDs come from small studies in restricted geographical areas.[13]

The costs of treatment, mainly long-term ones, can inflict further economic difficulties in populations already struggling to live with less than US$ 1 a day. Besides the obvious advantages of decreasing the healthcare costs due to lack of care or delayed care, investments in health improvement would also help to increase economic growth of the affected regions since healthier populations are more economically productive. [14–16]

As part of the movement to increase the attention given to NTDs, a coalition of many stakeholders gathered in January 2012 to discuss the importance of reaching the 2020 WHO goals for this group of diseases. As a result, the London Declaration was signed by many partners, committed to eradicate Guinea worm disease, eliminate three NTDs (lymphatic filariasis, leprosy, African sleeping sickness (human African trypanosomiasis) and blinding trachoma) and control the others (schistosomiasis, soil-transmitted helminths, Chagas disease, visceral leishmaniasis and river blindness (onchocerciasis)). [17,18]

A better understanding of the effect that NTD have on people’s economic livelihood would be an additional argument in favor of controlling or eliminating them. With this in mind, we performed a systematic literature review to identify and examine publications describing the impact of the London Declaration NTDs. Here we present the results for the five most prevalent ones, which are the ones eligible for preventive chemotherapy (PCT diseases): lymphatic filariasis, onchocerciasis, schistosomiasis, soil-transmitted helminths (ascariasis, trichuriasis, and hookworm infection) and trachoma on productivity loss in adults. [7,19,20]

## Methods

We performed a comprehensive search of the literature relating to the economic impact of all of the NTDs included in the London Declaration. Databases searched included Embase, Medline (OvidSp), Web of Science, Scopus, CINAHL, PubMed publisher, Cochrane, Popline, Lilacs, Scielo and Google Scholar. The search terms aimed at identifying articles about direct costs of treatment (such as consultation fees, medication, transport, food, assistance, accommodation), as well as indirect labor costs arising from decreased working hours and reduced economic activity attributable to morbidity. The search strategy included the names of the ten London Declaration NTDs (since many articles mention more than one) and words such as: ‘economic', 'financ', 'cost', 'productivity', 'absenteeism', 'employment', and 'cost'. A detailed list of the keywords used for each database is found in Supporting Information (S1 File). The search only considered title and abstracts, did not use any time restriction, and was restricted to the English language. The main database search was conducted in November 2013. There is no review protocol registered. This search included not only productivity loss, but also direct costs for all 10 London Declaration NTDs for a larger project. The results found in this article are limited to the results of the literature search regarding productivity loss from PCT NTDs.

The databases were merged according to the order shown in Table 1. Duplicates were removed automatically using Endnote and the remaining articles were then compared manually using author, year, title, journal, volume and pages to identify any additional duplicates. [21] After duplicates were excluded, we selected the articles that were related to each particular disease and screened the abstract and title of all papers to identify the ones that might provide information on productivity or indirect costs. The full-text versions of all remaining articles were then examined. Articles that did not contain any information on productivity, or only qualitative information on productivity loss (without any quantitative measures) were excluded, as well as articles that investigated productivity loss in children. Since the number of relevant publications was expected to be small, no restrictions were made regarding populations (participants), interventions, comparisons, outcomes, study design, or length of follow-up. Articles that could not be retrieved through their respective journals, contacting libraries, or after contacting the authors were classified as ‘not available’ and excluded from the selection. Any additional relevant articles identified when reading the full-text articles or checking their reference lists (i.e., the ‘snowball’ search strategy) were screened using abstract and title and then examined in more detail if they were considered potentially relevant.

**Table 1:** Results of database searches.

Database	Hits	After exclusion of duplicates
**Embase.com**	2913	2854
**Medline (OvidSP)**	2887	682
**Web-of-science**	1224	478
**Scopus**	3339	660
**CINAHL**	282	126
**PubMed publisher**	175	150
**Cochrane**	60	7
**Popline**	176	147
**Lilacs**	257	100
**Scielo**	36	26
**Google Scholar**	100	87
**Total**	11449	5316

In addition to searches using databases relating to the ‘white’ literature, we also searched the grey literature by screening websites of relevant organizations (i.e. World Health Organization, the Centre for Neglected Tropical Diseases, the Carter Center) (see S2 File). The list of selected articles for each disease was sent to disease experts identified in the literature and from institutions researching/combating NTDs, to check if the selection was comprehensive.

Data were extracted from selected articles independently, using a standardized Excel sheet, for the variables: author, year, study design, population, sample size, follow-up period, country, region, disease sequela, definition of productivity loss and results. Disease sequelae are disease manifestations, which for this review were defined by the Global Burden of Disease 2010 study (see S1 Table). [22] No summary measure was chosen beforehand. Instead, the results were presented separately per disease and study and described as they were reported in the articles; results were not statistically combined.

If the productivity loss was not already described in percentages of annual productivity in the articles, we calculated it whenever the unit of measurement made it possible, for the sake of comparability between studies and diseases. A working year was assumed to consist of 300 working days. [23]

Since the outcome of interest was productivity loss, various study designs were expected. The studies were therefore critically appraised regarding general criteria of selection, performance, attrition, detection, and reporting biases, as specified in the Cochrane Handbook for Systematic Reviews of Interventions. [24,25] Therefore, each article was given a rating regarding the risk of bias (possible options: low, high or unclear) for each criterion as well as a summary rating. [24,25] We added an extra criterion about the degree of relevance that the study outcomes defined as productivity loss had in terms of quantifying productivity loss in adults due to an NTD. This ‘relevance’ criterion was also rated as low or high. This review was conducted according to the PRISMA checklist for systematic reviews.

## Results

### Results of the database searches

Table 1 provides an overview of the databases searched and the number of articles identified through each of them. In total, 11,449 articles regarding all 10 NTDs were identified using the database searches. Of these, 5,316 articles remained after duplicates were removed. There was no duplication across the various NTDs.

#### Lymphatic filariasis

From the main database, 281 peer reviewed papers were related to lymphatic filariasis (LF). The grey literature search and snowballing method added 24 more articles, resulting in 305 articles being screened by title and abstract. Of the 72 full-text publications that were examined, 13 quantitatively described productivity loss related to LF (S1 Fig).

Lymphedema and hydrocele due to lymphatic filariasis are the two sequelae considered by the GBD study for this disease. Acute dermatolymphangioadenitis (ADLA) is part of these sequelae as acute inflammatory attacks suffered by most of the chronic patients, sometimes many times a year.[23]

An overview of the studies that used a quantitative method to describe productivity loss from lymphatic filariasis can be seen in Table 2, together with the calculated percentages of productivity loss.

**Table 2:** Description of studies investigating productivity loss due to lymphatic filariasis.

Author	Year	Country	Study design	Population	Sample size	Definition of productivity loss	Sequela	Results	Adjusted percentage of prod. loss ^1^
Babu	2002	India	Case vs control	Small farmers, daily wage laborers	377	Working hours/day	a) chronic filariasis (both sequelae)	a) 4.94 ± 3.33 vs 6.06 ± 3.22 controls	a) 18.48% (annual)
							b) lymphedema	b) male 4.45 ± 3.52 vs. 5.26 ± 3.21 controls (not significant) / female 5.45 ± 3.3 vs.7.12 ± 3.07 controls	b) male 15.40% (annual) / female 23.45% (annual)
							c) hydrocele	c) 4.98 ± 3.00 vs. 5.78 ± 2.84 controls	c) 13.84% (annual)
Babu	2003	India	Case vs control	Small farmers, daily wage laborers	1329	Hours spent in economic activity	ADLA	0.81 ± 2.31 h/day ADLA vs. 3.50 ± 3.74 h/day controls	76.85% (during ADLA episode) 1.58% (annual)
Babu	2006	India	Case vs control	Weavers	136	Hours spent in productive work/day	a) lymphedema	a) 8.02 ± 2.67 vs. 9.13 ± 1.61 controls	a) 10% (annual)
							b) hydrocele	b) 8.71 ± 1.86 vs. 10.08 ± 1.70 controls	b) 18.9% (annual)
Budge	2013	India	Pre/post-intervention	Homemakers/ Housekeepers	375	Working days lost to disability in the previous 30 days	Lymphedema	6.4 days	21.3% (annual)
Chandrasena	2004	Sri Lanka	Cohort	Patients attending morbidity control clinics	31	Capacity to perform any domestic or economic activity	ADLA	52% totally / 31.3% moderately incapacitated during ADLA episode	1.53% (annual)^2^
Chu	2010	Review	Review	Review	Review	Reduced work hours and economic activity	a) ADLA	a) 75% during ADLA episode	Idem
							b) lymphedema	b) 20%	b) 22.56% (annual)^3^
							c) hydrocele	c) 15%	c) 20.41% (annual)^3^
Gasarasi	2000	Tanzania	Cohort	Three villages in Rufiji district	65	Total incapacitation due to ADLA	ADLA	72.5% of the episodes, mean duration of 3.7 days	0.9% (annual)
Gyapong	1996	Ghana	Case vs control	Subsistence farmers	572	Ability to perform activities (vs others with similar diseases)	ADLA	at least 3 full days of incapacitation	at least 1% (annual)
Ramaiah	2000	India	Case vs control	Agricultural workers, carpenters, weavers	263 ADLA 478 Lym	Time spent on economic activity/day	a) ADLA	a) 0.97 ± 2.36 h/day vs. 4.48 ± 3.82 controls during attacks	a) 78.34% during ADLA episode
							b) lymphedema	b) 4.40 ± 3.79 h/day vs. 5.13 ± 3.83 controls	b)14.23% (annual), 24.3% (annual)^3^
Ramaiah	1999	India	Case vs control	Agricultural workers, carpenters, weavers	150	Time spent on economic activity/day	a) lymphedema	a) 3.93 h vs. 4.64 h controls	a) 15.3% (annual)
							b) hydrocele	b) 5.10 h vs 6.19 h controls	b) 17.6% (annual)
Ramaiah	1998	India	Case vs control	Two villages (south India)	124	Working hours	ADLA	0.68 ± 1.91h vs 4.40 ± 3.74 h controls / 3.58 ± 1.95 days duration/attack	84.54% during ADLA episode / 1% (annual)
Ramaiah	1997	India	Case vs control	Agricultural workers, weavers	372	Working hours	ADLA, lymphedema, hydrocele	28% worked fewer hours, 5% gave up work	^4^
Sabesan	1992	India	Case vs control	Patients attending filariasis clinics	528	Working days	ADLA	a) 23.4 days/year Bancroftian filariasis	a) 7.8% (annual)
								b) 26.5 days/year Brugian filariasis	b) 8.8% (annual)

ADLA—acute dermatolymphangioadenitis

1. Translation into percentage of productivity loss as described in the cited source, assuming 300 working days a year, for the ADLA episodes alone, for chronic sequelae alone, or for the weighted average of both, when applicable.

2. Totally incapacitated assumed 100% productivity loss, moderately incapacitated assumed 50% productivity loss during ADLA episodes

3. Weighted average including productivity loss from ADLA episodes and chronic symptoms

4. Only qualitative data, impossible to calculate annual productivity loss.

Productivity loss in LF patients can occur because of ADLA or the chronic sequelae of the disease (lymphedema and hydrocele). Our search identified six studies that examined only the acute attacks (ADLA), five articles that described the impact of chronic sequelae, and two that measured both.

The range in estimated productivity loss during ADLA attacks was 77–100% during the days of the attacks. The ranges in annual productivity loss reported in the literature were 10–26% for lymphedema and 15–19% for hydrocele (only the chronic sequelae). However, studies of productivity loss due to lymphedema and hydrocele rarely considered the different stages and varying severity of these symptoms. Most of the studies describing productivity loss due to LF measured it by comparing lost working hours or days amongst workers with LF with those seen amongst healthy workers.

#### Onchocerciasis

Of the 5316 articles in the source database, only 167 articles were related to onchocerciasis. In addition, 52 articles were found through the ‘snowball’ search and grey literature sources, which meant that a total of 219 articles were screened on abstract and title. Of these, 57 articles remained for full-text examination; from which only 10 contained quantitative information on productivity losses related to onchocerciasis (S2 Fig).

The GBD sequelae (disease manifestations) considered for onchocerciasis were skin disease and vision loss.

Table 3 provides an overview of studies that have quantitatively examined productivity loss resulting from onchocerciasis. Only one study—by Thomson—reported productivity loss due to onchocerciasis in general, of 20%.[26] The other papers focused on the effects of the specific sequelae of onchocerciasis on productivity.

**Table 3:** Description of studies investigating productivity loss due to onchocerciasis.

Author	Year	Country	Study design	Population	Sample size	Sequela	Definition of productivity loss	Results ^1^
Benton	1990	World	CBA/model	n/a	n/a	Blindness	Assumption	100%
Evans	1995	Guinea -OCP area	Observational (survey)	Household members in a highly endemic area	319	a) visual impairment	Self-reported 'inactive' occupational status	a) 38%
						b) blindness		b) 79%
Kim	1995	West Africa	CBA/model	n/a	n/a	Blindness	a) Productive years gained by preventing onchocerciasis blindness	a) 20 years
							b) Potential productivity loss	b) 100%
Kim	1997	Ethiopia	Case vs. control	Coffee plantation workers	235	a) OSD—intermediate	Daily wages (individuals infected with OSD vs. those without)	a) 10%
						b) OSD—severe	Daily wages (individuals infected with OSD vs. those without)	b) 15%
Okeibunor	2011	Cameroon, DRC, Nigeria, Uganda	Observational (cross sectional)	Primarily residents from villages where ivermectin distribution was ongoing	1600	General onchocerciasis	a) Increase in productivity from ivermectin treatment	a) 76%
							b) Percentage of respondents that referred ability to work better after ivermectin treatment	b) (75.6%)
Oladepo	1993	Nigeria	Case vs. control	Male farmers	102	OSD	Farm size that a men can keep satisfactorily weeded (workers with vs. without OSD)	9,117 vs 13,850 m^2^ (34% loss)
Thomson	1971	Cameroon	Case vs. control	Estate workers in an onchocerciasis endemic area	420	Unspecified (general)	Working days (workers with vs. without onchocerciasis)	20%
Wogu	2008	Nigeria	Observational (survey)	Rural farming community in a meso-endemic area	200	a) OSD—itching	a) Percentage of respondents that referred reduction in strength and concentration at work	a) 13.5%
						b) OSD—nodules	b) Percentage of respondents that referred decline in sales in business/trading	b) 11%
						c) visual impairment–ocular lesions	c) Percentage of respondents that reported giving up jobs (Productivity loss not specified)	c)14%
Workneh	1993	Ethiopia	Case vs. control	Male permanent coffee plantation workers	196	OSD	Absenteeism/sick leave and net monthly pay (workers with vs. without OSD)	25%
World Bank	1997	Nigeria, Ethiopia, Sudan	Case vs. control	Households in hyperendemic communities	824	OSD	Time spent on productive activities (individuals with vs. without OSD signs and symptoms)	not significant

CBA—cost-benefit analysis; OSD—onchocerciasis skin disease; n/a—not applicable

1. Percentage of annual productivity loss already calculated in the original publication

Four studies examined productivity loss related to onchocerciasis skin disease (OSD) [27–30]. Two of these studies compared Ethiopian coffee plantation workers with OSD to uninfected workers at the same plantation: Workneh et al.[29] concluded that workers with OSD had a one-year income that was 25% lower than that of healthy workers while Kim et al. [28] found 10–15% lower daily wages of individuals with OSD compared to those without. The study by Oladepo et al. [27] focused on the utilization of land and found that men with OSD had a significantly smaller (34%) amount of land than men without OSD. The study by the World Bank [30] found that individuals with onchocerciasis spent less time per day performing productive activities (farming and non-farming) and household activities than healthy individuals. However, these differences were not statistically significant.

Evans (1995) discussed the economic impact of blinding onchocerciasis [31], and found that visual acuity was strongly associated with occupational status. Approximately 80% of people that were blind due to onchocerciasis did not work, compared to 60% of the visually impaired (due to onchocerciasis) and 2% of the sighted.

Three studies (Thomson [26]; Wogu et al. [32] and Okeibunor et al. [33]) described in more general terms the socioeconomic consequences of onchocerciasis. For instance, Wogu et al. [32] reported that 13.5% of individuals with onchocerciasis-related itching experienced reduced concentration at work. In addition, 14% of the individuals with ocular lesion reported that they gave up their jobs because of visual impairment. Similarly, Okeibunor et al. [33] found that 76% of their subjects reporting increased productivity after (community based) treatment with ivermectin.

In addition to the observational studies of onchocerciasis-related productivity loss, we also identified several economic evaluations that considered productivity loss in their analyses. Two cost-benefit analyses, one by Benton and another by Kim, included productivity gains due to prevention of onchocerciasis blindness as part of the benefits of prevention. [34,35]. However, these gains were not actually observed in a patient population but based on the assumption that blind individuals are not productive at all. Kim et al. [35] assumed that each prevented case of blindness would result in 20 years of extra productivity.

#### Schistosomiasis

From the main search database, 670 articles referred to schistosomiasis, including publications identified through ‘snowball’ searching and grey literature sources. Of these, 26 articles were retrieved for full-text examination and eleven of them contained quantitative information on productivity losses caused by schistosomiasis (S3 Fig).

Three different worms of the genus Schistosoma can cause schistosomiasis: *Schistosoma haematobium*, *Schistosoma mansoni*, *and Schistosoma japonicum*. Ten sequelae were included for schistosomiasis in the GBD study: mild diarrhea, mild anemia, moderate anemia, severe anemia, hepatomegaly, hematemesis, ascites, dysuria, bladder pathology, and hydronephrosis due to schistosomiasis.

Table 4 provides a list of the studies investigating productivity loss attributable to schistosomiasis and the calculated percentages of annual productivity loss.

**Table 4:** Description of studies investigating productivity loss due to schistosomiasis.

Author	Year	Country	Study design	Population	Sample size	Sequela	Definition of productivity loss	Results	Percentage of annual prod. loss ^1^
Audibert	1998	Mali	Case vs. control	Families cultivating paddy in endemic region treated and not treated with praziquantel	412 households	infection by *S*. *haematobium; S*. *mansoni*	a) man-days worked/ha	a) 69 man-days per family worker	a) 23%
							b) farm size	b) additional 0.47 ha	b) 8.7%
Barbosa	1981	Brazil	Retrospective study + prospective study (both observational; matched case-control)	Sugarcane cutters; uninfected and stages I and 3 of a 3-stage clinical gradient (light, moderate, severe) for infected workers	94 (retrospective); 36 (prospective)	infection by *S*. *mansoni*	Reduced earnings compared to controls	Retrospective: no significant difference; Prospective: Stage III 31.9% to 38.4% less productivity vs. stage I	Idem ^2^
Blas	2006	Philippines	Observational study	Municipalities with relatively high endemicity	801	infection by *S*. *japonicum*	Loss of working capacity	Assumed loss: 25% (mild), 50% (moderate), 75% (severe), 100% (very severe)	Idem ^2^
Fenwick	1972	Tanzania	Observational study	Cane-cutters; men (uninfected, infected, treated)	approx. 300	*S*. *mansoni*	Mean bonus earnings, increase in cane cut	3% significant difference in productivity between uninfected and infected workers; true difference might be 5%	Idem ^2^
Kamel	2002	Egypt	Case vs. control	Textile company workers (infected vs healthy)	340 (170 vs 170)	infection by *S*. *haematobium; S*. *mansoni*	a) productivity score	a) 1059 vs 1113 (non-significant)	a) 4.8%
							b) additional hours/month	b) 26.5 vs 32.6 hours (significant)	b) 18.7%
							c) total incentives/month	c) 46.3 vs 56.2 incentives (significant)	c) 17.6%
Leshem	2008	Tanzania	Observational study	Israeli travelers	27	Acute schistosomiasis	# missed workdays	Average 7.8 days	2.6%
Leslie	2011	Niger	Cost effectiveness analysis	Schistosomiasis control programs (school-based vs community distributed MDA)	484	infection by *S*. *haematobium*	potential economic gain from adult treatment	$4.30, equal to 3 days of labor (based on agricultural day rate of $1.40 in 2005) or 2.3 days (based on rate of $1.90 in a normal year)	1%
Umeh	2004	Nigeria	Observational study	315 households from 4 communities	1763	Urinary Schistosomiasis	Average # of work-days lost due to urinary schistosomiasis	a) 4.7 days (head of household)	a) 1.5%
								b) 27.7 (adult male)	b) 9.2%
								c) 17.6 (wife)	c) 5.8%
								d) 24.7 (adult female)	d) 8.2%
								e) 19.09 weighted average	e) 6.3%
Wright	1972	Africa, Mauritius, Southwest Asia, Southeast Asia, America, World	Economic impact assessment	various	n/a	infection by *S*. *haematobium; S*. *mansoni; S*. *japonicum*	Reduced productive capacity	Assumed loss: 100% (severe), 10% (moderately severe)	Idem ^2^
Wu	2002	China	Case vs. control (matched)	Patients with advanced S. japonicum vs healthy individuals	48 cases, 56 controls	Advanced *S*. *japonicum*	Average workdays lost	Case vs. control: 4.11 vs. 0.86 days (p<0.01)	1%

n/a—not applicable; S. haematobium—*Schistosoma haematobium*; S. japonicum—*Schistosoma japonicum*; S. mansoni—*Schistosoma mansoni*, #—number of

1. Translation into percentage of annual productivity loss assuming 300 working days a year

2. Percentage of annual productivity loss already calculated in the original publication

The studies vary regarding schistosomiasis being caused by *S*. *haematobium*, *S*. *mansoni* or *S*. *japonica* and also regarding the sequela they focused on. Most of the studies we identified compared productivity loss between infected and uninfected workers in a company or municipality, whereas Blas et al. and Wright et al. calculated the costs of productivity loss based on assumptions and not on empirical data. [36,37] Productivity loss was also measured using different units: lost man-days/work days [38–41], reduced earnings/bonus/incentives [42–44], cane cut [45], and lost working hours [43].

#### Soil-transmitted helminths

In total, 538 articles in the source database were related to soil-transmitted helminths (STH)(ascariasis, trichuriasis and hookworm disease). The snowballing method and the gray literature search yielded an additional 48 articles, which meant that 586 articles were screened by title and abstract. Of the 72 publications that were fully read, only 6 had information related to productivity loss and STH (S4 Fig).

The GBD study lists the following sequelae related to each of the STH diseases: infestation, severe wasting, and mild abdominopelvic problems, as well as anemia only for hookworm disease.

Table 5 shows the list of studies, the summary of their findings regarding productivity loss as a consequence of STH, and the yearly percentages that were calculated wherever needed.

**Table 5:** Description of studies investigating productivity loss due to soil-transmitted helminths.

Author	Year	Country	Study design	Population	Sample size	Sequela	Definition of productivity loss	Results	Percentage of annual prod. loss ^1^
Basta	1979	Indonesia	Case vs control	Rubber plantation workers (male)	302	Anemia	a) collection of wet latex by tappers	a) 18.7% more	Idem ^2^
							b) removal of roots and weeds	b) 20% more	Idem ^2^
							c) physical capacity test (HST)	c) 15% higher HST scores in non-anemic group	Idem ^2^
Gilgen	2001	Bangladesh	RCT of iron-folic acid supplement & regular deworming	Tea pluckers (female) randomized to different treatment arms	553	Anemia	a) volume of green leaves plucked per day (kg/pld)	a) 1.8 less kg plucked (anemic vs. non-anemic)	a) 6.3%
							b) average wages earned per day	b) $1.1 less	b) 4%
							c) sick leave (# days)	c) 0.3 days more	c) 0.1%
							d) absenteeism (# days)	d) 0.9 days more	d) 0.3%
Selvaratnam	2003	Sri Lanka	Before-after	Tea pluckers (2500 m above sea level) (female)	304	Anemia	Volume of leaves plucked (per increase in hemoglobin of 1g/dL)	26% increase	Idem ^2^
Wolgemuth	1982	Kenya	Case vs control	Road construction workers (male, female)	47	Infection /Anemia	Volume of earth moved (m^3^/hour)	6% loss; Hb increase of 1.30 g/dL associated with a 5.6% increase in productivity	Idem ^2^
Casey	2011	Vietnam	Before-after (CEA)	Women in reproductive age in rural area	349	Anemia	Individual productivity gain after improvement of anemic state	5% in manual and 17% in heavy occupation (used values by Horton and Ross 2003)	Idem ^2^
Tanner	2013	Bolivia	Case vs control	Indigenous group of hunter-horticulturalists	86	Infection	Yield in agricultural and hunting/fishing (24h period)	(uninfected 6.8 kg vs. infected 4.4 kg; nonsignificant)	35.29%

CEA—Cost effectiveness analysis; RCT—randomized controlled trial, #—number of

1. Translation into percentage of annual productivity loss assuming 300 working days a year

2. Percentage of annual productivity loss already calculated in the original publication

Productivity loss from STH infection was generally measured by comparing infected to uninfected controls. Wolgemuth et al. observed that road construction workers with infection showed 6% less productivity (measured using volume of earth moved) than other workers. [46] Tanner et al. compared the agricultural and hunting or fishing yields reported in a 24-hour period among an indigenous Amazonian group of hunter–horticulturalists. There was a negative association of hookworm infection for both women and men, with hookworm-infected people reporting an average quantity of crops that was 35.29% less than uninfected people (no statistical significance). [47]

Productivity loss associated with anemia was mostly measured in women with three studies, by Casey et al., Gilgen et al., and Selvaratnam et al. [48–50] One study by Basta et al. investigated men [51] and one by Wolgemuth et al. investigated both women and men.[46] Two studies compared anemic versus non-anemic workers—Basta, and Gilgen [51,52], while three studies examined the productivity of the same individual twice, once while anemic and infected by STH and once after an intervention to increase the hemoglobin level—Casey, Selvaratnam, Wolgemuth [46,48,50], with or without deworming. The only randomized controlled trial was performed by Gilgen et al., assessing iron supplementation with and without deworming, with a significant negative association between hookworm infection and ferritin levels. Furthermore, anemic workers had a poorer performance regarding kilograms of leaves plucked and wages earned by day, as well as more sick and absent days compared to non-anemic workers. [52] There were two studies describing a positive linear association between hemoglobin and productivity. Selvaratnam et al found that an increase in 1g/dL in hemoglobin corresponded with an increase in 26% in a worker’s productivity. [50] Wolgemuth et al. described a linear increase in productivity ranging from 3.5% to 5.6% (depending on the formula used in the study) for each 1g/L in hemoglobin gain.[46]

#### Trachoma

In total, 538 articles from the initial search were related to trachoma and 11 articles were found through the ‘snowball’ search and grey literature sources, which led to a sum of 549 articles that were screened on title and abstract. Of these, 22 articles remained for full-text examination (S5 Fig).

The only sequela considered by the GBD study for trachoma was vision loss (from low vision to blindness).

A summary of the main features of the studies that investigated productivity loss due to trachoma quantitatively is shown in Table 6.

**Table 6:** Description of studies investigating productivity loss due to trachoma.

Author	Year	Country	Study design	Population	Sample Size	Sequela	Definition of productivity loss	Productivity loss ^1^
Frick	2001	The Gambia	Model	n/a	n/a	Low vision	Based on disability weight (GBD, 1996)	24.5%
Frick	2003a	Global	Model	n/a	n/a	a) blindness	Based on disability weight (GBD, 1996)	a) 60%
						b) low vision	Based on disability weight (GBD, 1996)	b) 24.5%
Frick	2003b	Global	Model	n/a	n/a	a) blindness	Assumptions based on disability weights (GBD, 1996), also assumed that 10% of blind persons required a caregiver who lost productivity completely	a) 60%/100%
						b) low vision	Assumptions based on disability weights (GBD, 1996), also assumed that 10% of blind persons required a caregiver who lost productivity completely	b) 24.5%

n/a—not applicable

1. Percentage of annual productivity loss actually used in the original publication

Of the studies we identified, none of them directly observed the extent of productivity loss caused by trachoma in a population. The three studies that examined this topic made assumptions about productivity loss in order to calculate the costs. [53–55] These studies assumed a productivity loss of either 60% or 100% for blindness and 24.5% for visual impairment and these percentages were based on the disability weights that existed at the time of the studies.

### Risk of bias

Sixty percent of the selected articles had a high overall risk of bias (26 articles of 42), mostly due to detection bias (24 of 42 articles), selection bias (21 articles of 42), and attrition bias (10 of 42 articles). Twenty-two articles were rated as relevant, and of these studies, two-thirds (14/21) had a high overall risk of bias, 2 had a low overall risk of bias and 6 had an unclear overall risk. Only 6 articles had a low overall risk of bias, of which only 2 were relevant, and 9 had an unclear summary rating, of which 6 were relevant (as described before). No particular trend was observed, regarding over- or underestimation of results due to bias. For the complete risk of bias assessment table, please refer to S3 Table.

## Discussion

Neglected tropical diseases can have a profound effect on the health and economic livelihood of the individuals suffering from them as well as that of their families. We examined what has been published in the literature regarding the loss in productivity seen amongst patients with the NTDs that are eligible for preventive chemotherapy: lymphatic filariasis, onchocerciasis, schistosomiasis, soil-transmitted helminths (ascariasis, trichuriasis, and hookworm infection) and trachoma. In general, our systematic literature review revealed that few studies have actually examined the degree of productivity loss related to these NTDs, which to some extent might have been influenced by the focus on literature written in English. Table 7 shows a summary of the flowcharts for all PCT NTDs, which shows the relatively small numbers of articles containing quantitative information on productivity loss related to PCT NTDs compared to the number of the publications screened by title and abstract.

**Table 7:** Summary of flowcharts for all PCT NTDs.

	Lymphatic filariasis	Onchocerciasis	Schistosomiasis	STH	Trachoma
Papers screened on title and abstract	305	219	670	586	549
Assessed full text	72	57	26	72	22
Studies with quantitative info on productivity losses	13	10	10	6	3

We also found large variation in the definition of productivity loss as well as the estimated productivity loss as reflected in percentage productivity loss over a one-year period. This is not surprising given the diversity in the methods chosen to quantify absolute and relative productivity loss, the many symptoms that these NTDs can cause, and the many different contexts of the different countries and regions where these diseases are endemic.

Many of these studies were performed many years ago and involved very specific populations in specific countries. However, besides biological reasons, there are methodological reasons for this variation. One explanation is simply random variation, where the results of two studies with the very same study design simply differ due to chance. A more important issue relates to the fact that studies varied in their approach when examining productivity loss. First of all, studies varied in their selection of the study population. Many studies focused on workers on large plantations, while others observed road workers, and the different studies were performed in different settings and countries, which might differ in important ways from other professions and other populations suffering from the same NTD elsewhere. The generalizability of the results from one study to another population must therefore be carefully considered.

The second type of variation in study design relates to the choice of comparison group. Most studies chose workers who did not have the NTD as the comparison group (only one, Gyapong 1996, compared patients with lymphatic filariasis with patients with other febrile diseases). The sometimes tacit assumption made with this comparison is that any difference in productivity can be attributed to the NTD and its symptoms. However, the validity of this comparison can be questioned, certainly if no correction is made for background characteristics such as age, sex, job experience, diet, height, and BMI (body mass index), which can affect productivity. Fortunately, some but not all studies included these factors when analyzing and reporting their results. Other studies did not compare two different populations but used a before-after study design to see how much productivity improved after treatment. This approach focuses directly on the productivity gain that can be achieved using available treatments.

The third type of variation relates to the actual measure of productivity. Many studies used the number of hours or days to quantify productivity. In contrast, some studies used other, arguably more accurate, methods which involved examining the volumes that were actually collected or processed per day (i.e. how many kilograms of tea were plucked per day). [49] Some studies even used multiple outcomes to study productivity loss. Ultimately, one could argue that the choice of outcome measure should be based on what a decision-maker considers important. For example, an employer might be particularly interested in volume outcomes since workers with an NTD who never miss a day at work may nevertheless be less productive than other workers. Another point worth considering when measuring productivity loss is that the adjustment of worker behavior and the associations between nutrition, body composition, and work productivity may be more complex. Workers might adapt work pace or intensity, allowing them to minimize the effects of poor health on work productivity. [47]

The fourth type of variation relates to the length of time that productivity loss is measured. Most studies used a fixed length of time (e.g., year) when measuring productivity, also to account for seasonality, which could also influence productivity along the year. In some instances, however, the length of time was disease-based (e.g., length of an episode). This approach may reveal that productivity loss is very high (>50%) if the symptoms are extreme, but the impact of disease on productivity over a longer period (e.g., one year) may be small if these episodes last just a few days and only occur a couple of times per year.

Other limitations of the studies are worth mentioning. Firstly, many studies did not check for other concomitant NTDs prevalent in the same region. One possible reason for this could be the assumption that the control group has the same risk to be affected by the non-investigated disease as the case group. Secondly, measurement of productivity loss in working populations may lead to an underestimation of impact due to the ‘healthy worker effect’, since people who had to stop working because of the disease are excluded from the study [38]. Thirdly. most of the studies that diagnosed NTDs using stool examination took only one sample, which resulted in a high probability of false negatives and a possible underestimation of productivity loss due to the NTD. [56–58] Lastly, correction of hemoglobin levels for altitude or for smoking status of the patients was not mentioned by any of the anemia studies, which could also lead to an underestimation of the productivity loss. [59]

Based on the literature, the NTDs with the greatest impact on an individual’s productivity loss are onchocerciasis and trachoma, because both of them can lead to blindness. The studies of actual patients revealed an increased likelihood of stopping with work or a substantial decrease in productivity. However, other studies simply assumed that productivity loss would be high.

It is important to distinguish between productivity loss at an individual level from productivity loss at a population level. For example, while the individual productivity loss from an NTD like STH may be much less than loss from another NTD like onchocerciasis, the overall impact of STH at a population level may be greater than that of onchocerciasis as a result of its higher prevalence. Therefore, what we consider important depends on the perspective we are taking (either that of the individual or that of the population).

The extent to which productivity is affected by diseases–in this case NTDs–can also help to understand the economic burden of diseases for affected individuals, countries, regions, and even globally. If we take the example of STH in India, around 50 million cases of hookworm (in adults older than 15 years) would be expected in 2020 if the epidemiological situation in 1990 had continued unabated. If we assume an annual income of US$1333 (which equals the annual income of an individual in the lowest GDP quintile in India in 2005) and an average productivity loss due to hookworm anemia of 6%, we could estimate an economic burden from productivity loss of roughly US$ 4 billion just in that one year. Obviously, the impact is much more pronounced when other years or countries are considered. These estimates can help to estimate the impact on productivity of achieving the targets described in the 2012 London Declaration. [18]

Some recommendations regarding future studies of productivity loss can also be made. The assessment of productivity loss secondary to NTDs should be further researched to enable a better understanding of the economic burden it generates. Additional research is needed to develop standard methods to describe absolute and relative productivity loss. However, this will not be an easy task, given the diverse symptoms caused by these diseases and the variety of countries and cultures where these diseases are endemic; with some NTDs such as lymphatic filariasis, a distinction between treated and untreated patients will have to be made as well. As described above, there are some factors that should be considered when designing future studies: the choice of the comparison group (preferably a comparable assuredly non-infected group), the outcome measure assessing productivity (preferably quantitative), the length of the assessment (not only during acute attacks, accounting for seasonal variation), and confounders of the disease effect on the productivity/work performance (for instance nutrition, BMI, type of work/profession). These elements should be transparently described and their (missing) values discussed, to determine how much they might have influenced the results. In particular, researchers should provide sufficiently detailed information to enable readers to assess the quality and relevance of the study.

### Conclusion

Various studies have examined productivity loss in patient populations having one of the five most prevalent NTDs. While is clear that these diseases reduce productivity, the actual impact depends on the type, severity and duration of the NTD as well as on the setting. Variation in estimated productivity loss between and within diseases is caused by differences in the different definition of productivity loss, research methods and setting. It is therefore important to examine the literature carefully to understand what was actually observed in order to draw conclusions about the generalizability of the studies. Since productivity loss is an important aspect of the burden of diseases, further research on better estimates of the magnitude of the productivity loss caused by NTDs would enable a more complete picture of their economic burden to individuals, countries, and globally, adding an additional persuasive argument in favor of their control.

This review already contributes to a better perception of the magnitude of the effect of an NTD on people’s working and economic situation, and can already offer additional arguments in favor of controlling and eliminating them. However, there is still much room for further research in this field to improve the understanding on NTDs’ effects on individuals’ productivity loss.

## Supporting Information

S1 File Literature search syntax. (PDF)

S2 File Grey literature search. (PDF)

S1 Table List of disease sequelae according to the 2010 GBD study. (PDF)

S2 Table Risk of bias assessment table. To which extent it measured productivity loss caused by NTD quantitatively. CBA—Cost-benefit analysis. CEA–Cost-effectiveness analysis. OBS–Observational study. RCT–Randomized controlled trial.
(PDF)

S3 Table PRISMA checklist. (PDF)

S1 Fig Flowchart describing the literature search for lymphatic filariasis. (TIF)

S2 Fig Flowchart describing the literature search for onchocerciasis. (TIF)

S3 Fig Flowchart describing the literature search for schistosomiasis. (TIF)

S4 Fig Flowchart describing the literature search for soil-transmitted helminths. (TIF)

S5 Fig Flowchart describing the literature search for trachoma. (TIF)
