# Supplementary material for: Productivity Loss Related to Neglected Tropical Diseases Eligible for Preventive Chemotherapy: A Systematic Literature Review
Source: PLoS Negl Trop Dis. 2016 Feb 18;10(2):e0004397. doi: 10.1371/journal.pntd.0004397 (PMC4758606; doi:10.1371/journal.pntd.0004397)
Supplement: S2 File — (PDF) [file pntd.0004397.s002.pdf]

S2. Grey Literature Search

The publications of the following organizations were searched: BMGF, Carter Center, CBM International (Christian Blind Mission), CDC Centers for Disease Control and Prevention, Centre for Neglected Tropical Diseases (Liverpool School of Tropical Medicine), Drug for Neglected Diseases Initiative, Global Alliance to Eliminate Lymphatic Filariasis, Global Network - NTD (Sabin Vaccine Institute), Hellen Keller International, Hollows, IDA Foundation, IDB - Interamerican Development Bank, IMA World Health, IMF, Imperial College London, International Trachoma Initiative, Lepra, Liverpool Associates in Tropical Health, PLOS NTD x economic, Research Triangle Institute – RTI, Sightsavers, The Task Force for Global Health, UN, USAID, WHO, World Bank, World Vision.
