# Supplementary material for: Productivity Loss Related to Neglected Tropical Diseases Eligible for Preventive Chemotherapy: A Systematic Literature Review
Source: PLoS Negl Trop Dis. 2016 Feb 18;10(2):e0004397. doi: 10.1371/journal.pntd.0004397 (PMC4758606; doi:10.1371/journal.pntd.0004397)
Supplement: S1 Table — (PDF) [file pntd.0004397.s003.pdf]

S3. List of disease sequelae according to the 2010 GBD study

Disease	Sequelae GBD	Sequalae
Onchocerciasis	Skin disease due to Onchocerciasis	Mild skin disease due to Onchocerciasis
		Moderate skin disease due to Onchocerciasis
	Vision loss due to Onchocerciasis	Blindness due to Onchocerciasis
		Low vision due to Onchocerciasis
		Visual field impairment due to Onchocerciasis
		Visual field blindness due to Onchocerciasis
Lymphatic Filariasis	idem	Lymphedema
	idem	Hydrocele due to lymphatic filariasis
STH - Ascariasis	idem	Ascariasis infestation
	idem	Severe wasting due to ascariasis
	idem	Mild abdominopelvic problems due to ascariasis
STH -Trichuriasis	idem	Trichuriasis infestation
	idem	Severe wasting due to trichuriasis
	idem	Mild abdominopelvic problems due to trichuriasis
STH - Hookworm disease	idem	Hookworm infestation
	idem	Severe wasting due to hookworm disease
	idem	Mild abdominopelvic problems due to hookworm disease
	Anemia due to hookworm disease	Mild anemia due to hookworm disease
		Moderate anemia due to hookworm disease
		Severe anemia due to hookworm disease
Schistosomiasis	idem	Schistosomiasis
	idem	Mild diarrhea due to Schistosomiasis
	Anemia due to Schistosomiasis	Mild anemia due to Schistosomiasis
		Moderate anemia due to Schistosomiasis
		Severe anemia due to Schistosomiasis
	idem	Hepatomegaly due to Schistosomiasis
	idem	Haematemesis due to Schistosomiasis
	idem	Ascites due to Schistosomiasis
	idem	Dysuria due to Schistosomiasis
	idem	Bladder pathology due to Schistosomiasis
	idem	Hydronephrosis due to Schistosomiasis
Trachoma	Trachoma	Low vision due to trachoma
		Blindness due to trachoma
HAT	idem	African trypanosomiasis
Chagas disease	idem	Acute chagas disease
	idem	Chronic heart disease due to chagas disease
	idem	Chronic digestive disease due to chagas disease
	idem	Heart failure due to chagas disease
Leishmaniasis	idem	Visceral leishmaniasis
	idem	Cutaneous leishmaniasis
Leprosy	idem	Disfigurement due to leprosy
