# Supplementary material for: Productivity Loss Related to Neglected Tropical Diseases Eligible for Preventive Chemotherapy: A Systematic Literature Review
Source: PLoS Negl Trop Dis. 2016 Feb 18;10(2):e0004397. doi: 10.1371/journal.pntd.0004397 (PMC4758606; doi:10.1371/journal.pntd.0004397)
Supplement: S2 Table — To which extent it measured productivity loss caused by NTD quantitatively. CBA—Cost-benefit analysis. CEA–Cost-effectiveness analysis. OBS–Observational study. RCT–Randomized controlled trial. (PDF) [file pntd.0004397.s004.pdf]

S9. Risk of bias assessment table

Author	Year	Study design	Selection bias	Performance bias	Attrition bias	Detection bias	Reporting bias	Summary risk	Relevance[1]
**Lymphatic filariasis**									
Babu	2002	OBS	Unclear	Low	Low	High	Unclear	High	High
Babu	2003	OBS	High	Low	Low	High	Low	High	High
Babu	2006	OBS	High	Low	Low	High	Low	High	High
Budge	2013	OBS	Unclear	Low	Low	Low	Low	Low	Low
Chandrasena	2004	OBS	High	Low	Low	High	Low	High	Low
Gasarasi	2000	OBS	Unclear	Low	Low	High	Low	High	Low
Gyapong	1996	OBS	High	Unclear	Low	High	Unclear	High	Low
Ramaiah	2000	OBS	High	Low	High	High	Low	High	High
Ramaiah	1999	OBS	High	Low	Low	High	Low	High	High
Ramaiah	1998	OBS	High	Low	Unclear	High	Low	High	High
Ramaiah	1997	OBS	High	Unclear	Unclear	High	High	High	Low
Sabesan	1992	OBS	High	Unclear	High	High	Unclear	High	High
**Onchocerciasis**									
Benton	1990	CBA/model	Low	Unclear	Unclear	High	Low	High	Low
Evans	1995	Survey	Low	Low	Unclear	Unclear	Low	Unclear	Low
Kim	1995	CBA/model	Low	Low	Unclear	High	Low	High	Low
Kim	1997	OBS	High	Low	Low	Unclear	Unclear	Unclear	High
Okeibunor	2011	OBS	Low	Low	Unclear	Low	Low	Low	Low
Oladepo	1993	OBS	Low	Unclear	Unclear	Unclear	Low	Unclear	High
Thomson	1971	OBS	High	Unclear	High	High	High	High	High
Wogu	2008	Survey	Low	Unclear	Low	Unclear	Low	Unclear	Low
Workneh	1993	OBS	High	Low	Unclear	Unclear	Low	Unclear	High
World Bank	1997	OBS	High	Unclear	Unclear	Unclear	Unclear	Unclear	High
**Schistosomiasis**									
Audibert	1998	OBS	Low	Low	Low	Unclear	High	Unclear	High
Barbosa	1981	OBS	Low	Low	Low	High	High	High	High

Blas	2006	OBS	High	Unclear	High	Unclear	Unclear	High	Low
Fenwick	1972	OBS	High	Unclear	High	Unclear	Low	High	High
Kamel	2002	OBS	High	Unclear	High	High	Unclear	High	High
Leshem	2008	OBS	Low	Low	Low	Low	Low	Low	Low
Leslie	2011	CEA	High	Unclear	High	High	High	High	Low
Umeh	2002	OBS	Low	Low	Unclear	low	low	Low	Low
Wright	1972	Economic impact	High	Unclear	Unclear	High	Low	High	Low
Wu	2002	OBS	Unclear	Low	Unclear	Low	Low	Low	High
**Soil-transmitted helminths**									
Basta	1979	OBS	High	Unclear	High	Unclear	High	High	High
Gilgen	2001	RCT	Unclear	Unclear	Unclear	Unclear	High	Unclear	High
Selvaratnam	2003	OBS	Unclear	Low	Low	Unclear	Low	Low	High
Wolgemuth	1982	OBS	Unclear	High	High	High	Unclear	High	High
Casey	2011	CEA	High	Low	High	High	High	High	Low
Tanner	2013	OBS	High	Unclear	Unclear	High	Low	High	High
**Trachoma**									
Frick	2001	Model	Low	Low	Unclear	High	Low	High	Low
Frick	2003a	Model	Unclear	Unclear	Unclear	High	Low	High	Low
Frick	2003b	Model	Unclear	High	Unclear	High	Low	Unclear	Low
