# Supplementary material for: Productivity Loss Related to Neglected Tropical Diseases Eligible for Preventive Chemotherapy: A Systematic Literature Review
Source: PLoS Negl Trop Dis. 2016 Feb 18;10(2):e0004397. doi: 10.1371/journal.pntd.0004397 (PMC4758606; doi:10.1371/journal.pntd.0004397)
Supplement: S3 Table — (PDF) [file pntd.0004397.s005.pdf]

PRISMA 2009 Checklist

Productivity Loss Related to Neglected Tropical Diseases Eligible for Preventive Chemotherapy: a Systematic Literature Review

Section/topic	#	Checklist item	Reported on page #
**TITLE**			
Title	1	Identify the report as a systematic review, meta-analysis, or both.<br>**Productivity Loss Related to Neglected Tropical Diseases Eligible for Preventive Chemotherapy: a Systematic Literature Review**	1
**ABSTRACT**			
Structured summary	2	Provide a structured summary including, as applicable: background; objectives; data sources; study eligibility criteria, participants, and interventions; study appraisal and synthesis methods; results; limitations; conclusions and implications of key findings; systematic review registration number.	2
**INTRODUCTION**			
Rationale	3	Describe the rationale for the review in the context of what is already known.	Pages 3 and 4
Objectives	4	Provide an explicit statement of questions being addressed with reference to participants, interventions, comparisons, outcomes, and study design (PICOS).	
**METHODS**			
Protocol and registration	5	Indicate if a review protocol exists, if and where it can be accessed (e.g., Web address), and, if available, provide registration information including registration number.<br>'There is no review protocol registered.'	4
Eligibility criteria	6	Specify study characteristics (e.g., PICOS, length of follow-up) and report characteristics (e.g., years considered, language, publication status) used as criteria for eligibility, giving rationale.<br>'Since the number of relevant publications was expected to be small, no restrictions were made regarding populations (participants), interventions, comparisons, outcomes, study design, or length of follow-up.'	4
Information sources	7	Describe all information sources (e.g., databases with dates of coverage, contact with study authors to identify additional studies) in the search and date last searched.<br>(information on white and grey literature sources)<br>'The list of selected articles for each disease was sent to disease experts identified in the literature and from institutions researching/combating NTDs, to check if the selection was comprehensive.'	4 and 5
Search	8	Present full electronic search strategy for at least one database, including any limits used, such that it could be repeated.<br>(all search strategies are fully presented in the Supporting Information)	S1.Literature Search Syntax
Study selection	9	State the process for selecting studies (i.e., screening, eligibility, included in systematic review, and, if applicable, included in the meta-analysis).	4

		‘Articles that did not contain any information on productivity, or only qualitative information on productivity loss were excluded, as well as articles that investigated productivity loss in children.(…) Articles that could not be retrieved through their respective journals, contacting libraries, or after contacting the authors were classified as ‘not available’ and excluded from the selection.	
Data collection process	10	Describe method of data extraction from reports (e.g., piloted forms, independently, in duplicate) and any processes for obtaining and confirming data from investigators.<br>(see item 11)	5
Data items	11	List and define all variables for which data were sought (e.g., PICOS, funding sources) and any assumptions and simplifications made.<br>‘Data were extracted from selected articles independently, using a standardized Excel sheet, for the variables: author, year, study design, population, sample size, follow-up period, country, region, disease sequela, definition of productivity loss and results.’	
Risk of bias in individual studies	12	Describe methods used for assessing risk of bias of individual studies (including specification of whether this was done at the study or outcome level), and how this information is to be used in any data synthesis.<br>‘Since the outcome of interest was productivity loss, various study designs were expected. The studies were therefore critically appraised regarding general criteria of selection, performance, attrition, detection, and reporting biases. Each article was given a rating for low, high or unclear risk of bias for each criterion and a summary rating. (1,2) We added an extra criterion to assess to which extent the study outcomes defined as productivity loss were relevant when describing quantitative work productivity loss in adults due to an NTD. This ‘relevance’ criterion was also rated as low or high.’	5
Summary measures	13	State the principal summary measures (e.g., risk ratio, difference in means).<br>‘No summary measure was chosen, the results were presented separately per disease and per study, descriptively (results were not statistically combined).’	5
Synthesis of results	14	Describe the methods of handling data and combining results of studies, if done, including measures of consistency (e.g., I^2) for each meta-analysis.<br>(see item 13)	5

Section/topic	#	Checklist item	Reported on page #
Risk of bias across studies	15	Specify any assessment of risk of bias that may affect the cumulative evidence (e.g., publication bias, selective reporting within studies).	Not applicable
Additional analyses	16	Describe methods of additional analyses (e.g., sensitivity or subgroup analyses, meta-regression), if done, indicating which were pre-specified.	Not applicable
**RESULTS**			
Study selection	17	Give numbers of studies screened, assessed for eligibility, and included in the review, with reasons for exclusions at each stage, ideally with a flow diagram.	5 to 18 (flow diagrams per

Study characteristics	18	For each study, present characteristics for which data were extracted (e.g., study size, PICOS, follow-up period) and provide the citations.	disease shown in Supporting Information)
Risk of bias within studies	19	Present data on risk of bias of each study and, if available, any outcome level assessment (see item 12).	18 and Supporting Information 4
Results of individual studies	20	For all outcomes considered (benefits or harms), present, for each study: (a) simple summary data for each intervention group (b) effect estimates and confidence intervals, ideally with a forest plot.	
Synthesis of results	21	Present results of each meta-analysis done, including confidence intervals and measures of consistency.	Not applicable
Risk of bias across studies	22	Present results of any assessment of risk of bias across studies (see Item 15).	Not applicable
Additional analysis	23	Give results of additional analyses, if done (e.g., sensitivity or subgroup analyses, meta-regression [see Item 16]).	Not applicable
**DISCUSSION**			
Summary of evidence	24	Summarize the main findings including the strength of evidence for each main outcome; consider their relevance to key groups (e.g., healthcare providers, users, and policy makers).	18 and 19
Limitations	25	Discuss limitations at study and outcome level (e.g., risk of bias), and at review-level (e.g., incomplete retrieval of identified research, reporting bias).	19 to 21
Conclusions	26	Provide a general interpretation of the results in the context of other evidence, and implications for future research.	
**FUNDING**			
Funding	27	Describe sources of funding for the systematic review and other support (e.g., supply of data); role of funders for the systematic review.	Described during submission

From: Moher D, Liberati A, Tetzlaff J, Altman DG, The PRISMA Group (2009). Preferred Reporting Items for Systematic Reviews and Meta-Analyses: The PRISMA Statement. PLoS Med 6(6): e1000097. doi:10.1371/journal.pmed1000097

For more information, visit: **www.prisma-statement.org**.
