# Supplementary figures and images for: Productivity Loss Related to Neglected Tropical Diseases Eligible for Preventive Chemotherapy: A Systematic Literature Review
Source: PLoS Negl Trop Dis. 2016 Feb 18;10(2):e0004397. doi: 10.1371/journal.pntd.0004397 (PMC4758606; doi:10.1371/journal.pntd.0004397)

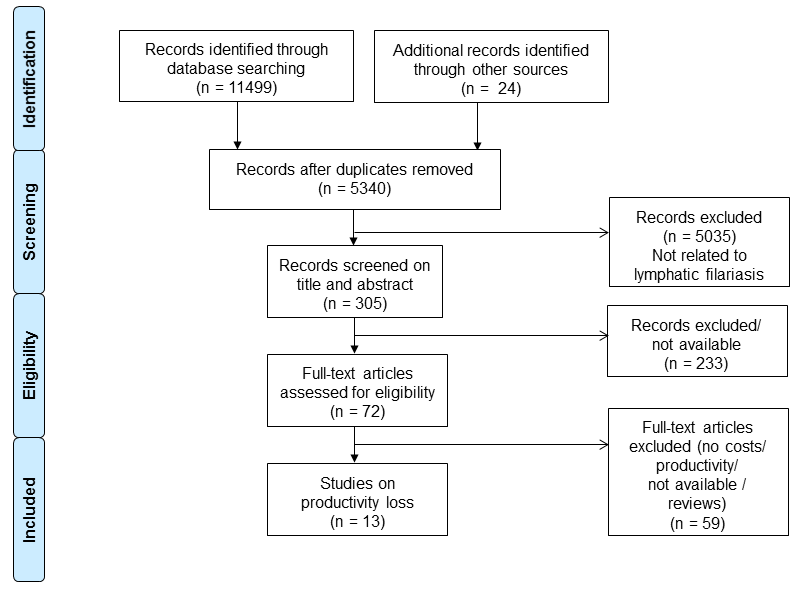

Supplement: S1 Fig — (TIF) [file pntd.0004397.s006.tif]

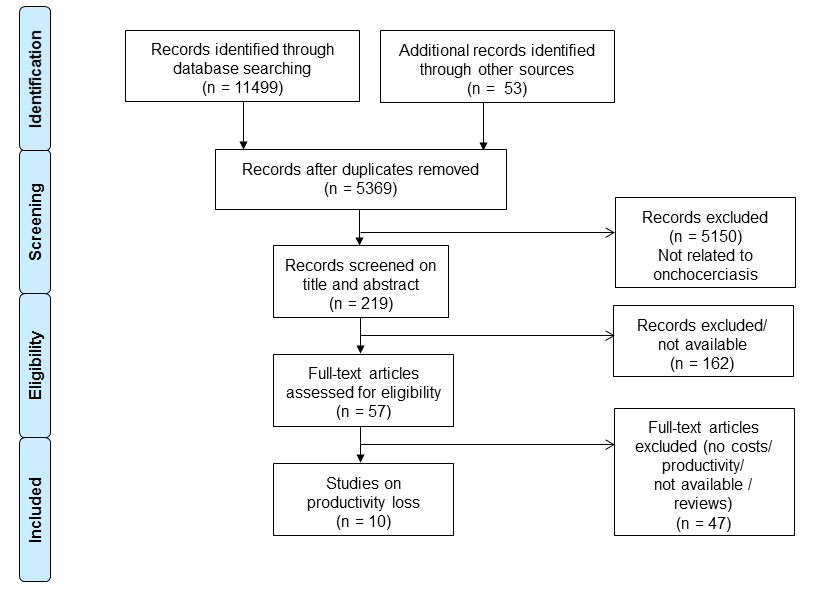

Supplement: S2 Fig — (TIF) [file pntd.0004397.s007.tif]

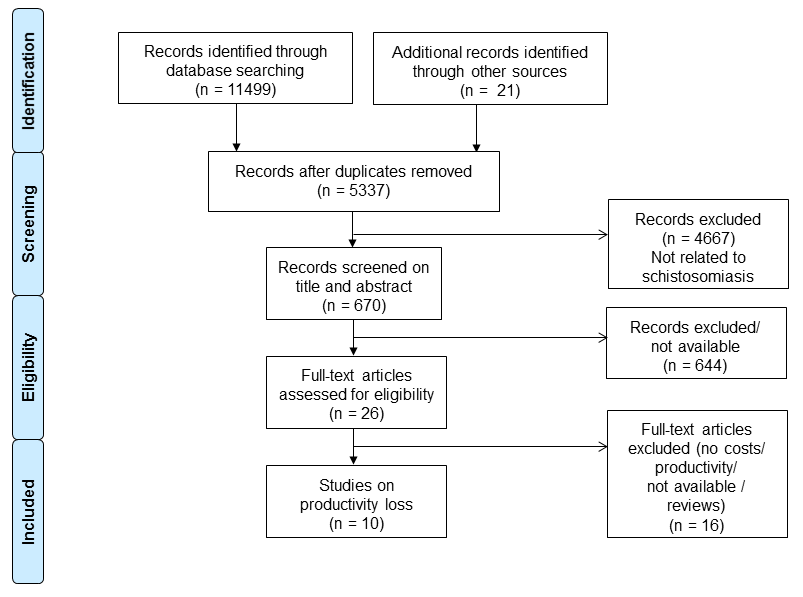

Supplement: S3 Fig — (TIF) [file pntd.0004397.s008.tif]

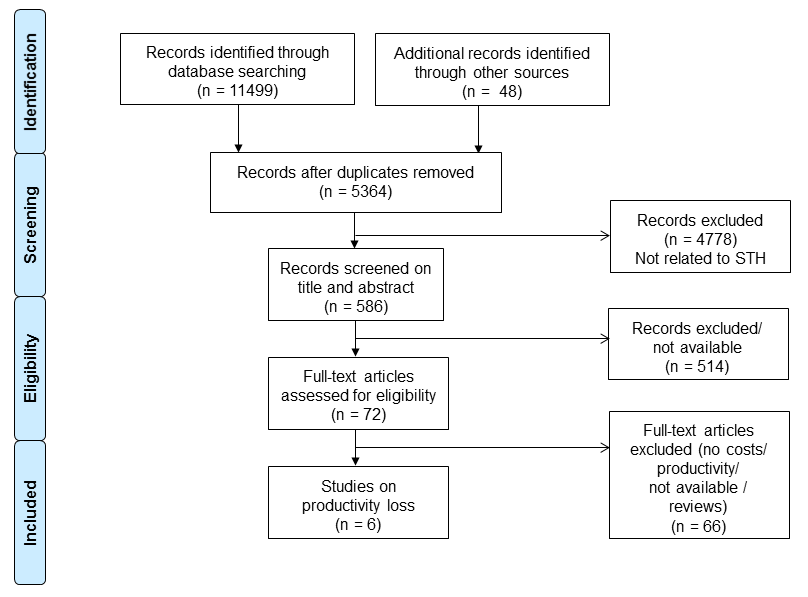

Supplement: S4 Fig — (TIF) [file pntd.0004397.s009.tif]

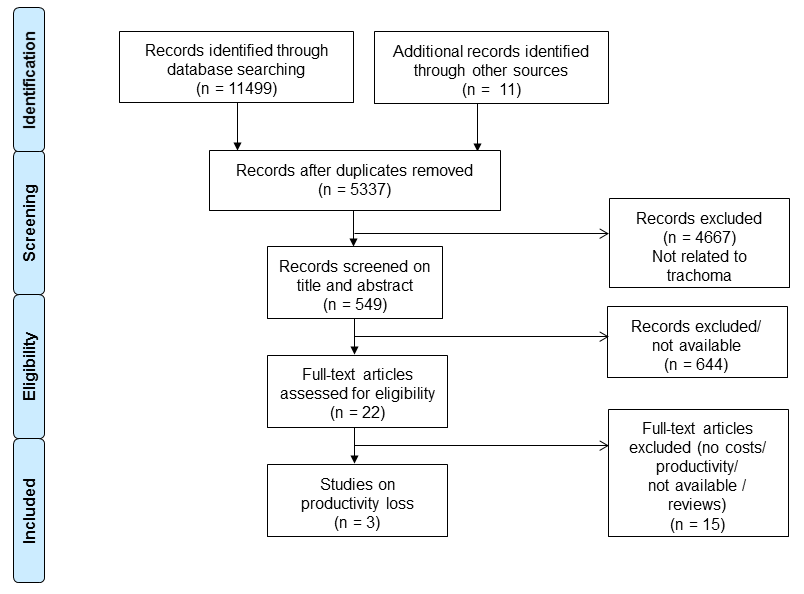

Supplement: S5 Fig — (TIF) [file pntd.0004397.s010.tif]
